# Comparative toxicological evaluations of novel forms nano-pesticides in liver and lung of albino rats

**DOI:** 10.1007/s10735-023-10115-y

**Published:** 2023-03-31

**Authors:** Abeer M. Abdel-Azeem, Eman S. Abdel-Rehiem, Ahmed A. Farghali, Fatma K. Khidr, Manal Abdul-Hamid

**Affiliations:** 1 Cell Biology, Histology and Genetics Division, Department of Zoology, Faculty of Science, Beni-Suef University, P.O. BOX 62511, Beni-Suef, Egypt; 2 Molecular Physiology Division, Zoology Department, Faculty of Science, Beni-Suef University, P.O. Box 62511, Beni-Suef, Egypt; 3 Materials Science and Nanotechnology Department, Faculty of Postgraduate studies for Advanced Sciences, Beni-Suef University, P.O. Box 62511, Beni-Suef, Egypt; 4 Animal Research Department, Plant Protection Research Institute, Agricultural Research Center, Cairo, Egypt

**Keywords:** CuONSp, CuONF, Liver toxicity and lung toxicity, NF-kβ, P53, Histopathological and ultrastructural studies

## Abstract

Copper oxide Nanoparticles (CuONPs) are used in different agricultural applications. Large amounts of CuONPs cause organ dysfunction in animals. Our study aim to compare between the toxic effects of CuONanSphere (CuONSp) and CuONanoFlower (CuONF) as new nano-pesticides, determine a less toxic form when used in agricultural applications. To characterize CuONSp and CuONF, we used X-ray diffraction (XRD), Field emission scanning electron microscopy (SEM), and High resolution transmission electron microscopy (HRTEM) and Zeta-sizer device.18 adult male albino rats were divided into three groups (n = 6), (I) control group, (II) and (III) groups were given orally 50 mg/kg/day of CuONSp and CuONF 30 days respectively. CuONSp induced oxidant-antioxidant abnormalities, including an increase in malondialdhyde (MDA) and a decrease in glutathione (GSH) in comparison to CuONF-treated one. CuONSp induced an increase in liver enzymes activities compared to CuONF. Tumour necrosis factor-alfa (TNF-α) detected an increased in liver and lung compared to CuONF. However, histological examinations revealed changes in CuONSp group than CuONF group. Changes in immune-expressions of TNF-α, nuclear factor kappa-light-chain-enhancer of activated B cells (NF-kβ) and tumour suppressor gene (p53) were also more identified in CuONSp group than CuONF group. Ultrastructural studies of liver and lung tissues marked alternations were observed in CuONSp group than CuONF group. In conclusion, CuONSp induced biological alternation in liver and lung more than CuONF. So, CuONF is less toxic compared to CuONSp when used as nano-pesticide in agricultural applications.

**Graphical abstract:**

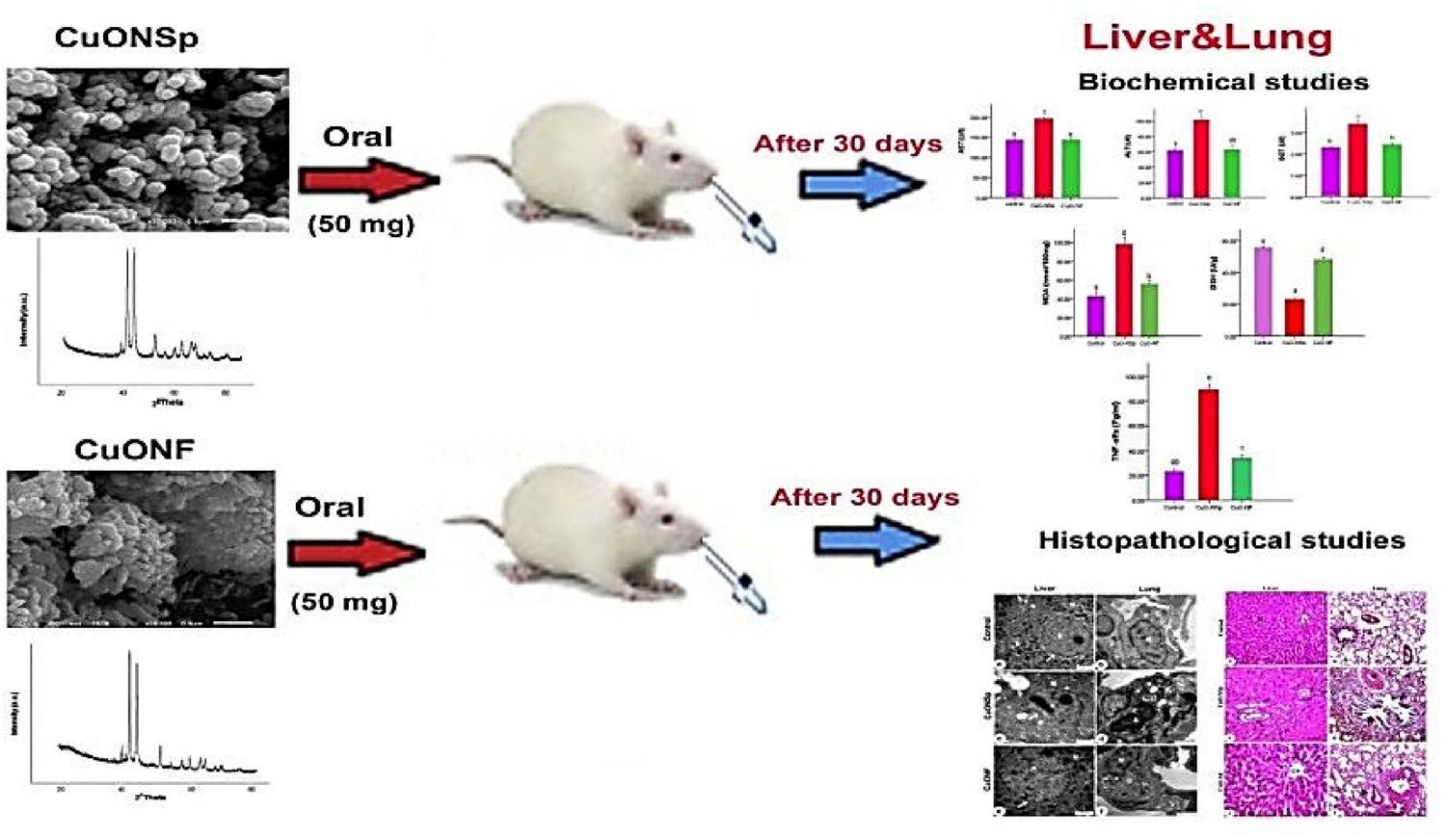

**Supplementary Information:**

The online version contains supplementary material available at 10.1007/s10735-023-10115-y.

## Introduction

NPs has a lot of uses in different applications, because of their unique physicochemical characteristics such as changing their size, surface properties, and shape in application-specific ways (Aillon et al. 2009). CuO NPs have been proposed for use in soils as fertilisers, fungicides, and pesticides in order to increase agricultural sustainability (Pelegrino et al. 2020). CuO nanostructures with a variety of morphologies have been synthesised, including nanospheres, nanorods, nanoneedles, nanoflowers and nanotips. The CuO nanostructures were synthesised using a variety of methods, ranging from microwave to hydrothermal synthesis (Zhou et al. 2006). Nonetheless, their unique physicochemical characteristics can enhance their toxicological effect in vivo by promoting particle cell uptake and translocation through blood circulation (Kang et al. 2013). In vitro investigations on human cells exposed to CuO NPs revealed high cytotoxicity as well as the ability to produce DNA damage, oxidative stress, and cell death (He et al. 2020).

The physiological environment has a significant effect on NPs metabolic pathways, which will affect their existence and biosecurity (Yallapu et al. 2015). The extensive usage of NPs has raised concerns about the health dangers and environmental problems that come with their use (Johnson et al. 2015). Furthermore, the absence of biosafety assessments limited the use of NPs (Oberdörster et al. 2005).

The decrease in blood antioxidant activities caused by CuO NP exposure reveals the severity of the oxidative stress detected (Tulinska et al. 2022). The toxicity of NPs and the excessive growth of reactive oxygen species (ROS) have been related to oxidative stress, and caused apoptosis could be one of the NPs possible toxicity pathways (Benameur et al. 2015). The activities of both ALT and AST increased in blood serum. The release of liver enzymes into the blood serum is considered as an indication of liver disease or histological toxic damage (Miron and Mahbubeh 2014). The glutathione peroxidase (GPX) activity decreased in cells exposed to CuONPs. CuONPs decrease GPX activity, indicating that they not only create free radicals but also effect on the antioxidant cell resistance (Fahmy and Cormier 2009). Overproduction of ROS disrupts the balance of the liver’s oxidative and antioxidant processes, resulting in an increased in lipid peroxidation by MDA and caused hepatocyte death (Brown et al. 2004). The serum level of TNF-α was increased in CuONPs treated rats (Abdelazeim et al. 2020).

The semipermeable, discontinuous endothelium of the hepatic sinusoids and blood arteries, which allows circulating NPs of less than 100 nm to enter the hepatic parenchyma by opsonisation, may be linked to the extensive bioaccumulation of CuONPs in the liver (Sadauskas et al. 2007). The cytotoxicity of liver caused by CuONPs may involve autophagy or cell egodigestion, which is a conserved mechanism involved in the breakdown of proteins and organelles by the cytoplasm (Baehrecke 2005). CuO NPs toxicity has been linked to oxidative stress and CuO propensity to destroy mitochondria in liver cells (Karlsson et al. 2009). CuONPs are similarly exceedingly toxic, also, causing severe pulmonary edoema and mortality (Yokohira et al. 2009). CuONPs also induced apoptosis in adenocarcinomic lung epithelial cell line in lung tissues in a dose-dependent manner (Xiaofeng et al. 2018). CuONPs revealed alterations in the lung, such as edematous expansion of membranous pneumocytes and endothelial cells in some areas (Dumkova et al. 2016). Cells are known to reverse the overwhelming oxidative stress response through increased expression of cytokines such as TNF-α, kinase activation, and phosphatase inhibition, thus affecting the cascade of phosphorylation (Genestra 2007). Several metal oxide NPs increase the activation of NF-kβ pathways (Smith et al. 2001). Expressions of the p53 protein level up-regulated on exposure to CuONPs (Gopinath et al. 2010).

In the previous studies, liver and lung toxicity was induced by CuO-Nanosphere, but CuONF not used in biological applications. So, the present study aims to compare between the toxicological effects of CuONSp and CuONF as a new nano-pesticides, since no studies performed on the CuONF form to know the less toxic form which can be applied in agriculture field.

## Materials and methods

### Method of CuONanSphere (CuONSp) synthesis

We used the hydrothermal method in synthesis of CuONSp. Cu (NO_3_)_2_.3H_2_O and NaOH were dissolved in pure water and then transferred to an autoclave under steady stirring. The autoclave was kept at 170 ºC for 24 h. The autoclaves were brought to room temperature by being cooled in the air. The recovered precipitates are centrifuged, washed several times with pure water and ethanol, and then dried for 24 h in a drying oven at 60 ºC (Wang et al. 2014).

### Method of CuONanoFlower (CuONF) synthesis

We used the hydrothermal method in synthesis of CuONF. In deionized water, CuCl_2_ was dissolved. The CuCl_2_ solution was then gently added to the NaOH solution, while stirring vigorously, yielding a blue-colored precursor. The blue precursor has been introduced to cetyl trimethyl ammonium bromide (CTAB) and forcefully stirred to ensure complete dissolution of CTAB. This reaction solution was then transferred to autoclave with and heated in an electric oven120 °C for 6 h. After the reaction, the autoclave was allowed to cool to room temperature. The dark precipitate was centrifuged and washed thoroughly with deionized water and ethanol. The precipitate was then dried in a drying oven for 24 h in a drying oven at 60 °C (Zou et al. 2011).

### Characterizations of CuONSp and CuONF

#### 1-X-ray diffraction (XRD) measurements of CuONSp and CuONF

X-ray diffraction (XRD) patterns were used to investigate the crystalline structure of CuONSp and CuONF. At room temperature, the XRD pattern of two shapes of CuO NPs was obtained using a PAN alytical X’Pert X-ray diffractometer fitted with a Ni filtered Cu Kα (λ = 1.54056 ºA) radiations as the X-ray source. The measurements were carried out with 2θ range 10 < θ < 80 with step size 0.04.

#### 2-SEM and HRTEM measurements

In this study, samples were measured by SEM (JSM5610LA/ Japan) and HRTEM (JEM2100/ Japan) at Beni-Suef University to study the morphology and size of CuONSp and CuONF.

#### 3-Nano-measurements by the Zeta-sizer device

The average hydrodynamic size, PDI and zeta potential of CuONSp and CuONF in double distilled water were estimated using dynamic light scattering (DLS) (Nano-Zeta-Sizer-HT, Malvern Instruments, Malvern, UK) at room temperature (Murdock et al. 2008).

### Experimental animals

Eighteen Wistar male albino rats weighing 120–150 g were used in this study. They were purchased from the Egyptian Organization for Biological Vaccine Production (A.R.E.). They were housed in stainless steel cages at room temperature (25 ^°^C) and on a natural light/dark cycle, with complete nutrition pellets and water available at all times. Prior to the start of the experiment, all of the animals were isolated for ten days. All animals were isolated for 10 days prior to the start of experiment. The study was approved by the Ethics Committee of the Beni-Suef University (BSU-IACUC, No. 019–78).

### Animals grouping

Animals were assigned into three groups (n = 6); the first group (G1) was the untreated control group, the second (G2) and third groups (G3) were received 50 mg/kg/day of CuONSp and CuONF respectively for 30 days according to Arafaa et al. (2017). At the end of experiment the animals were sacrificed.

### Biochemical studies

#### Blood sampling

At the end of the treatment, blood samples were taken from rats from various groups and blood samples were taken in tubes (Halperim et al.^1951^).

#### Liver functions

The activities of AST, ALT, and GGT were measured at the end of the experiment method in serum (Schumann and Klauke 2003). The DxC uses an enzymatic rate technique to determine GGT activity in serum. The rate of change in absorbance is proportional to the amount of GGT activity in the sample (Beckman et al. 2007).

### Oxidative stress and TNF-α measurements

Lipid peroxidation is measured as MDA. The amount of MDA in the liver and lung was measured according to Ohkawa et al. (1979) procedure. The activity of GPX in hepatic and pulmonary was investigated according to Beutler et al. (1963) process. Rat TNF-ELISA Kit (CSB-E11987r) was used to measure TNF-α activity in the liver and lung.

### Histological preparations

At the end of treatment, animals from each group were anaesthetized with light diethyl ether and dissected to remove the liver and lung for histological preparations. Parts 4 to 5 μm thick were made using a microtome and stained with haematoxylin and eosin for histological examinations, according to Bancroft and Gamble (2002).

### Immunohistochemical and morphometric study

Other sections of liver and lung were mounted on + ve charged slides for immunohistochemical inspection. The sections put in 3% H_2_O_2_ followed by citrate buffer. The sections were probed with an antibody against TNF-α, NF-kβ and p53 then washed in phosphate buffer and incubated with the secondary antibody. The sections were counter-stained with Mayer^,^s haematoxylin. We used Leica Qwin 500 LTD image analysis (Cambridge UK) in our morphometric measurements. The area % of TNF-α, NF-kβ and p53 immunoreactivity was recorded in immuno-stained sections. The measurements were done in 10 random high powers (X400) nono-overlapping fields for each section using the binary mode.

### Ultrastructural preparations

Slices of liver and lung tissue were fixed in 3% glutaraldhyde- formaldhyde, pot-fixed in osmium-tetroxide according to Bozzola and Russell (1999). The ultra-microtome glass knives were then used to cut ultrathin sections, which were dyed with uranyl acetate and lead citrate (Reynolds 1963) then was viewed at an accelerating voltage with a Joel CX 100 transmission electron microscope (TEM).

### Statistical analysis

The Statistical Package (SPSS for WINDOWS, version 20.0; SPSS Inc, Chicago) was used in the social sciences (IBM Corp, 2011). Results were expressed as mean ± standard error and values of P > 0.05 were considered non-significantly different, while those P < 0.05 and P < 0.01 were significant and high significant differentiation, respectively.

## Results

Our study illustrates a comparison study between the toxicological effects of CuONSp and CuONF on the liver and lung of male albino rats.

### X-ray diffraction (XRD) measurements of CuONSp and CuONF

**Fig. 1:**
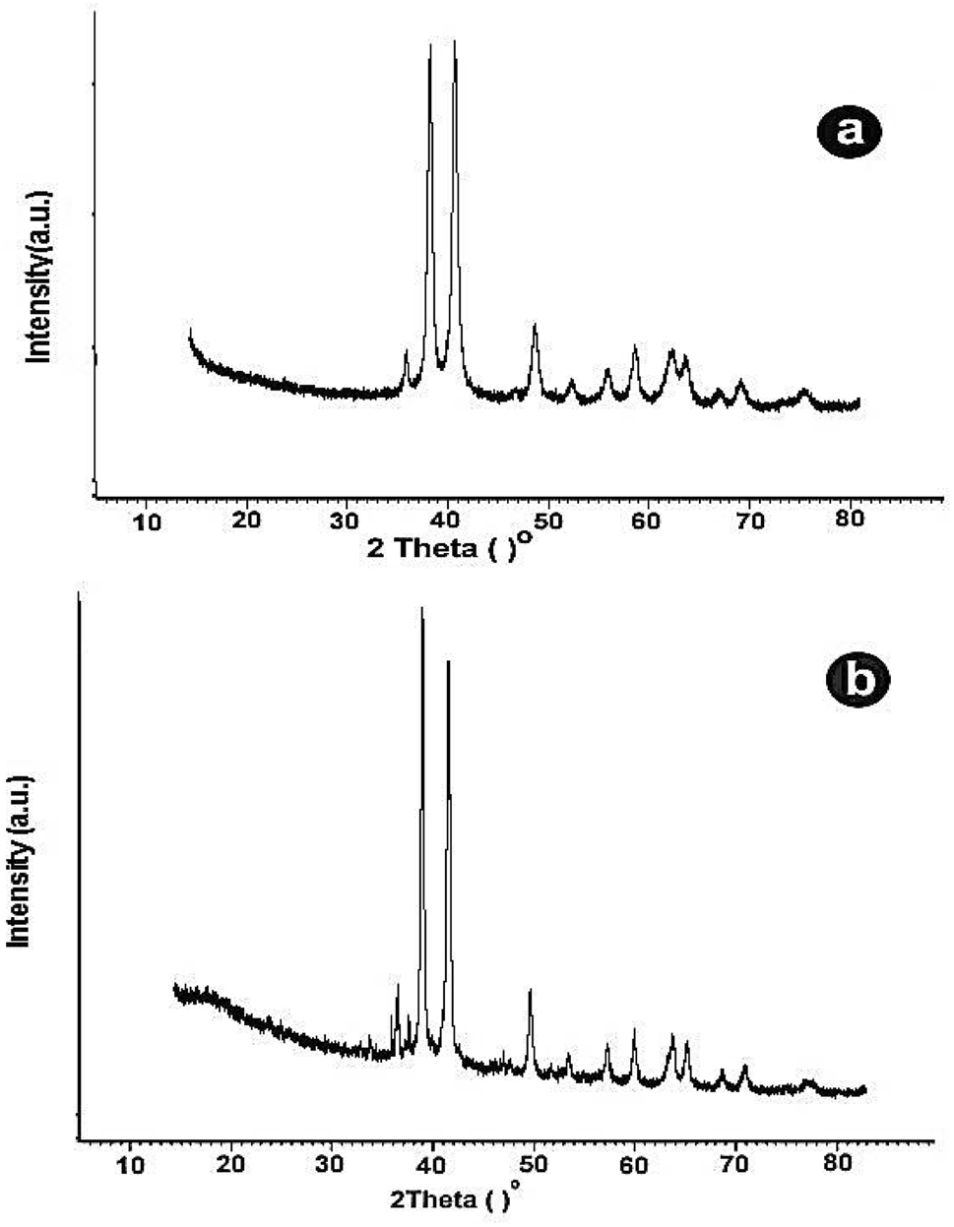
X-ray diffraction patterns of **a** CuONSp and **b** CuONF.

The diffraction patterns of the prepared nanostructures are illustrated in (Fig. 1a, b). The XRD patterns of (Fig. 1a) were compared and indexed with the standard JCPDS file number 96-901-5925.The data revealed the formation of CuO nano structures in monoclinic symmetry with Space group C12/c1 (Table S1). In Fig. 1b the XRD patterns were compared and indexed with the standard JCPDS file number 96-901-5823. Also, the data revealed the formation of CuO nano structures in monoclinic symmetry with Space group C12/c1 (Table S2). No extra peaks were observed pointing to the single plane structure.

The crystallite size of the samples under investigation was computed using Debye schetter formula (Ahmed et al. 2016) L$=\frac{k\lambda }{\beta COS \theta }$ ; where k is the shape factor, λ is the target wave length; β is the corrected full width of half maximum and θ is the diffraction angle. The CuONSp was found to be 20.6 nm. While the CuONF was117.1 nm.

### SEM and HRTEM measurements

**Fig. 2:**
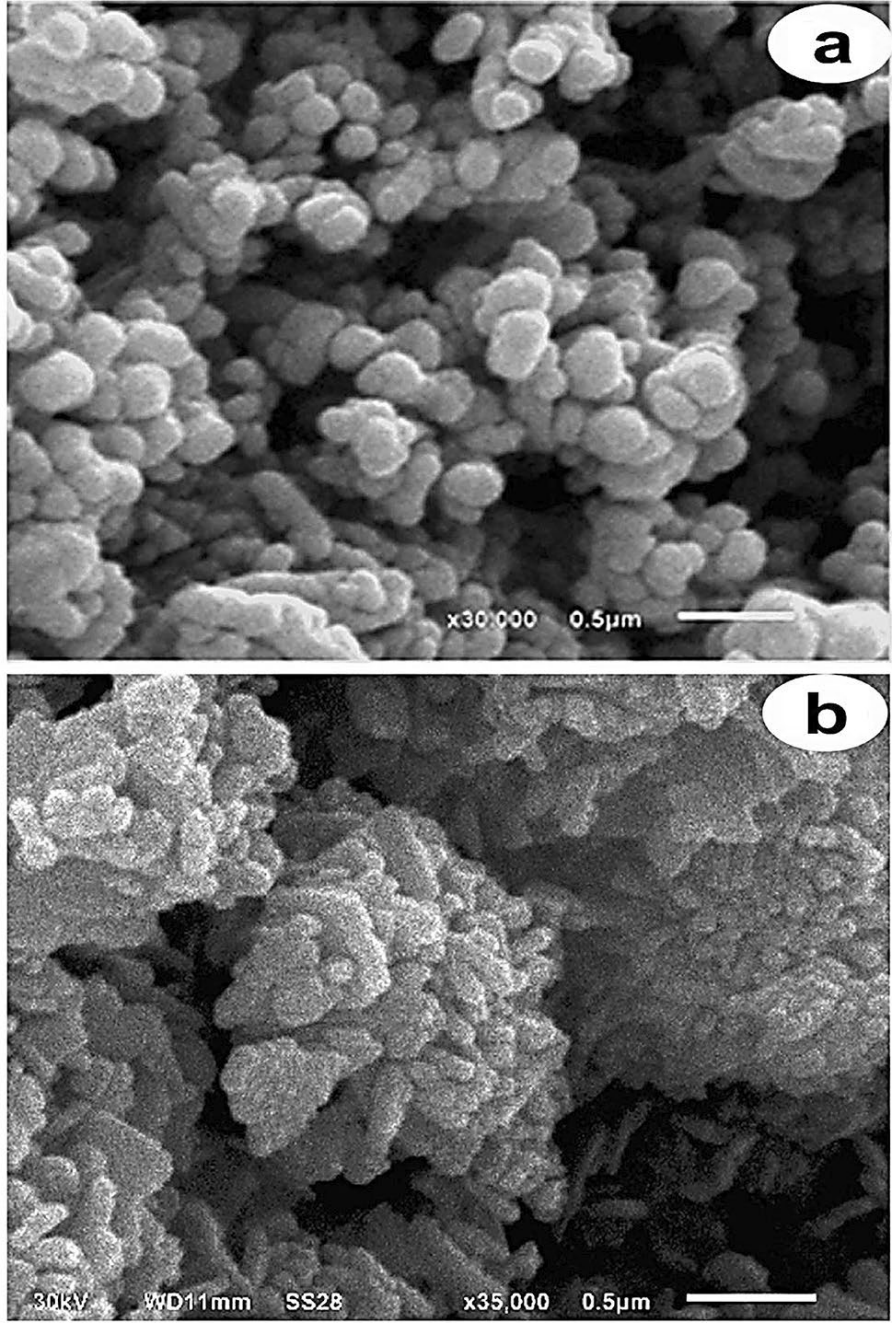
Scanning electron microscope (SEM) images of **a** CuONSp and **b** CuONF

To describe morphological and microstructural features to CuONSp and CuONF. We used SEM to reveal the presence of agglomerated spherical grains in CuONSp (Fig. 2a) and agglomerated grains in CuONF (Fig. 2b). Modifying the shape of the surface reduced the amount of interaction between NPs and cells. The more the shape surface of NPs is modified, the less contact with cells. As a result, we expected CuONSp has higher toxicity than CuONF.

**Fig. 3:**
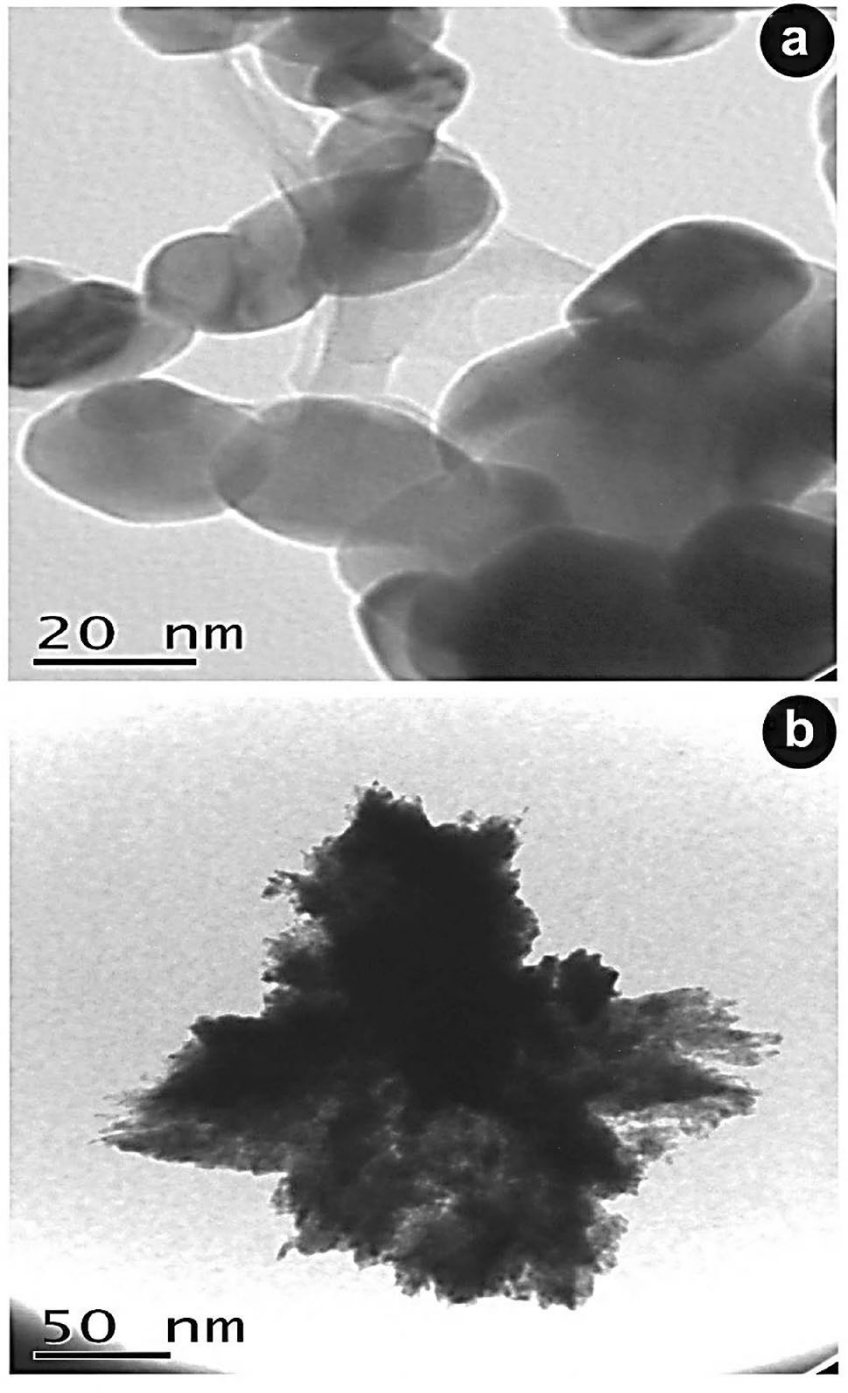
High resolution transmission electron microscope (HRTEM) images of distinct forms of CuONSp (**a**) and CuONF (**b**)

From HRTEM the size of CuONSp was observed range of 9 nm (Fig. 3a), while, CuONF was measured at range 228 nm (Fig. 3b). One of the key factors contributing to the toxic impact of NPs was their size. The smaller in nanosize, the higher in toxicity, and as seen in the previous data, CuONSp nanosize is smaller than CuONF. Therefore, CuONSp is expected to higher toxicity than CuONF.

### Nano-measurements by the Zeta-sizer device

**Fig. 4:**
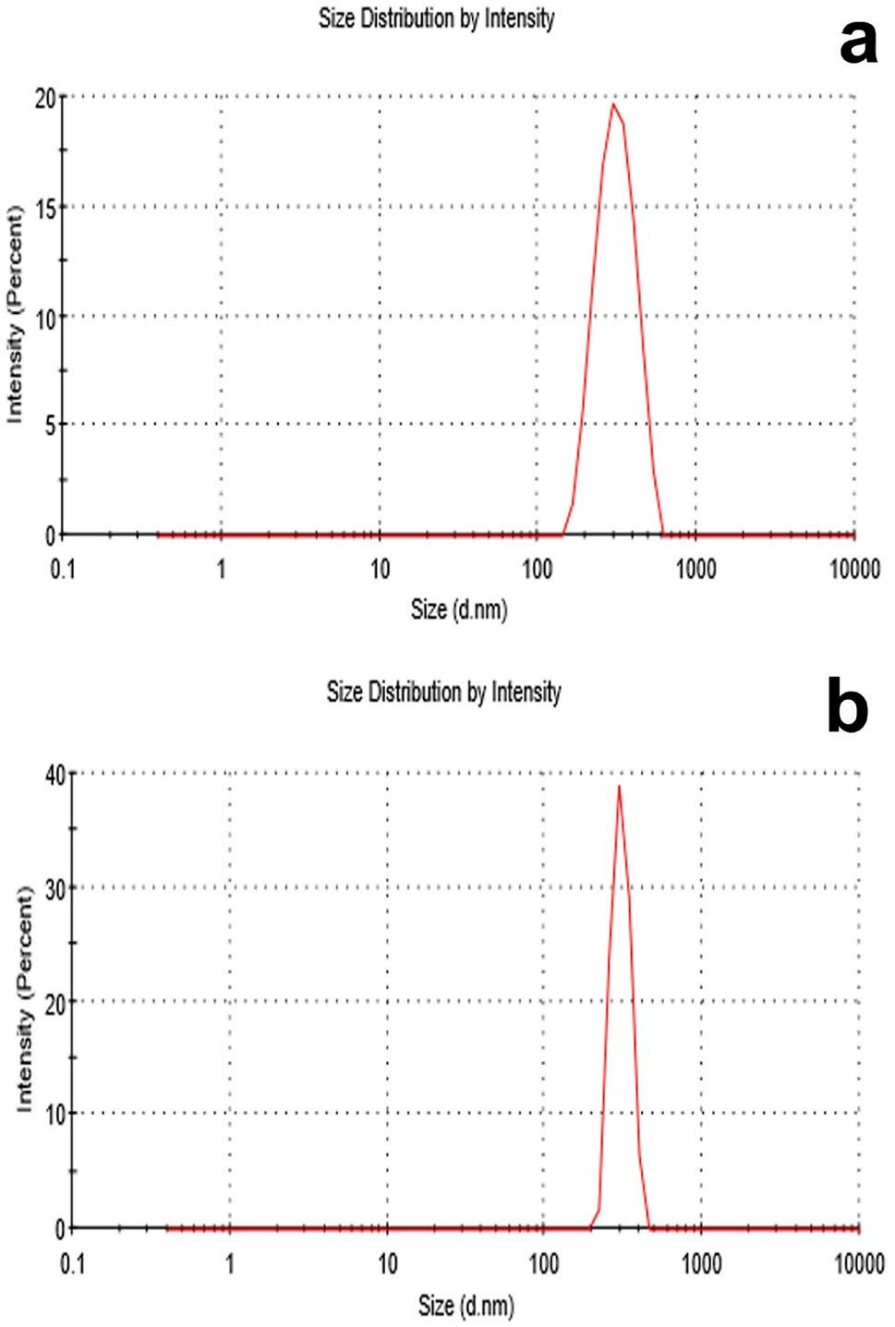
Hydrodynamic size distribution of CuONSp (**a**) and CuONF (**b**)

The hydrodynamic size of two different shapes in doubled distilled water was measured in CuONSp at range 284.7 ± 59.7 d.nm, (Fig. 4a) and in CuONF at range 363 ± 82.6 d.nm (Fig. 4b). As a result, we expected the toxicity of CuONSp to be higher than CuONF, due to its small size.

The polydispersity index (PDI) was used to determine NPs stabilityin suspension.The PDI in doubled distilled water was found in CuONSp at 0.43 and in CuONF at 0.64, indicating a stable suspension. The PDI illustrates NPs size distribution, a lower value indicates more particle stability and more toxicity. Therefore, it appears that the CuONSp is more widely distributed and harmful than CuONF.

**Fig. 5:**
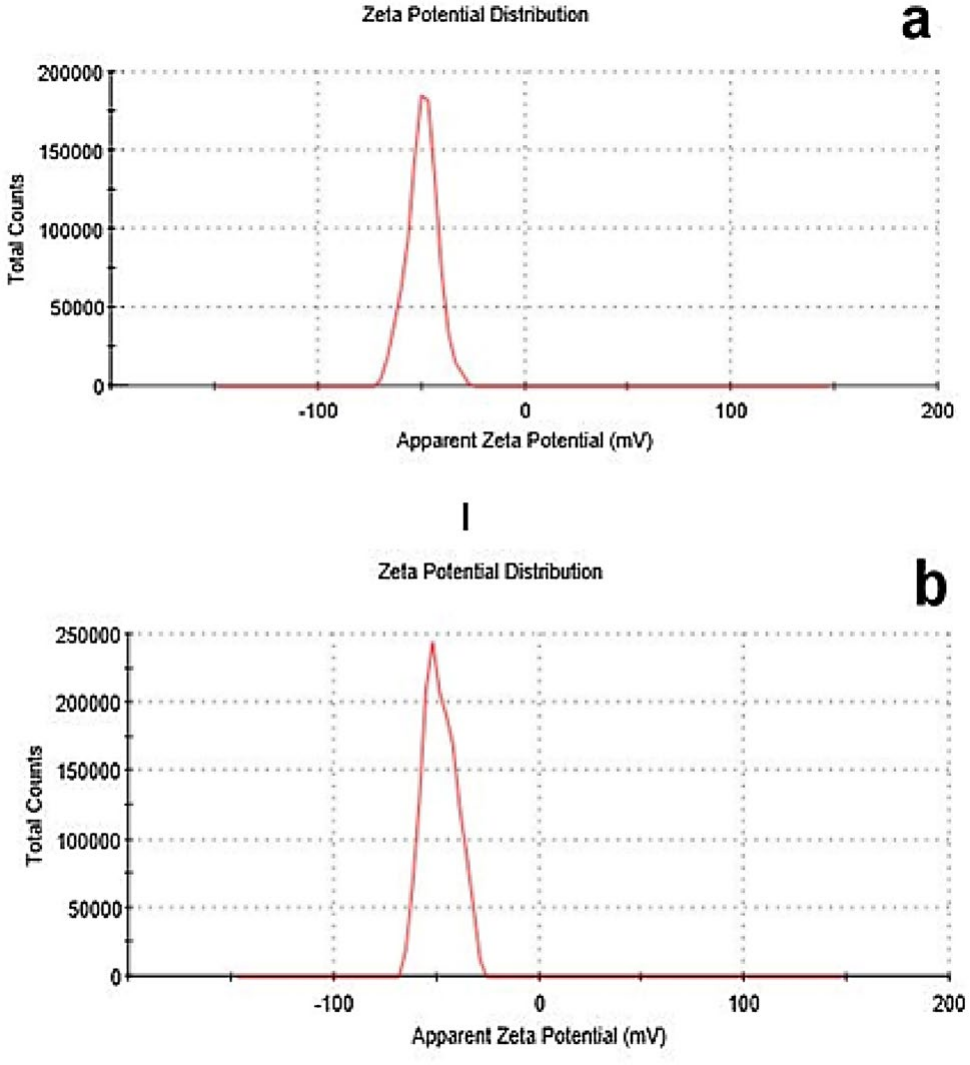
Zeta potential distribution of CuONSp (**a**) and CuONF (**b**)

Zeta Potential of CuONSp in doubled distilled water measured at − 50.9 ± 6.5 mV (Fig. 5a) and in CuONF at range − 47.2 ± 7.6 mV (Fig. 5b). As a result, CuONSp has more charges than CuONF. The interactions of NPs with biological systems are determined by their surface charge. More toxicity was caused by a significant amount of surface charge. As a result, we believed CuONSp is more toxic than CuONF.

### Biochemical effects of CuONSp and CuONF on liver functions, oxidative stress and TNF-α

**Fig. 6:**
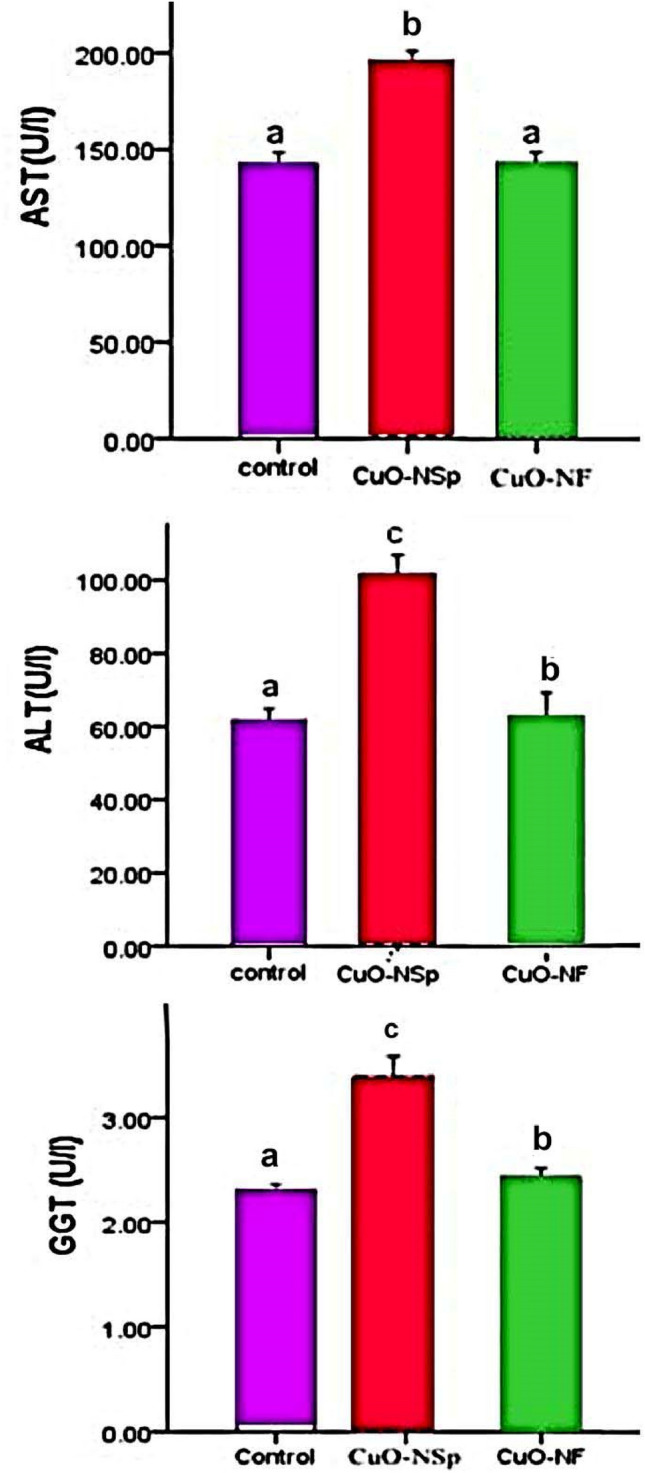
The toxic Effects of CuONSp and CuONF on Serum AST, ALT and GGT activities at the end of experiment. ^a,b,c^ indicated the difference or similarity between groups. Group with different superscript letters are considered significantly different (P < 0.05). There are six animals in each group

CuONSp induced increase in serum AST activity with high significant (*P <* 0.01) (194.6 ± 6.3U/l), and CuONF at 144.2 ± 5.5 U/l compared to normal control ones (142.1 ± 6.2 U/l) at the end of experiment (Fig. 6). Also, CuONSp induced an elevation in serum ALT activity with high significant (*P <* 0.01) (101.8 ± 5.2U/l) and CuONF measured at 63.2 ± 6.6U/l compared to normal control ones (61.7 ± 3.1U/l) at the end of experiment. In addition, CuONSp induced arise in serum GGT activity with high significant (*P <* 0.01) (3.4 ± 0.18U/l) and CuONF at 2.4 ± 0.1 U/l compared to normal control rats (2.29 ± 0.1 U/l) at the end of experiment. As above result, the elevation of AST, ALT & GGT activities in CuONSp more than CuONF compared to control group (Fig. 6). This mean CuONSp induced more toxicity on liver function more than CuONF.

**Fig. 7:**
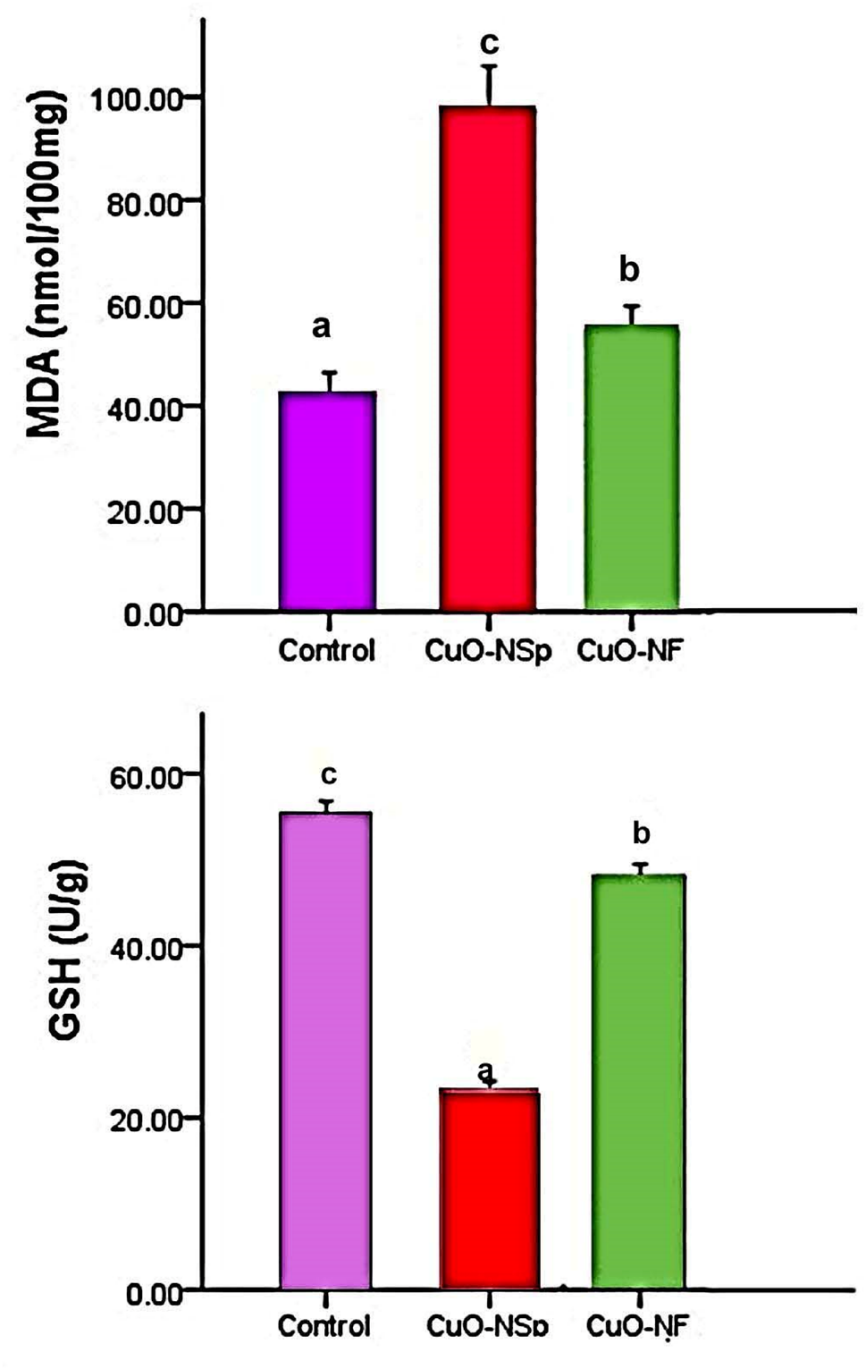
The toxic effect of CuONSp and CuONF on MDA level and GSH activity at the end of experiment. ^a,b,c^ indicated the difference or similarity between groups. Group with different superscript letters are considered significantly different (P < 0.05). There are six animals in each group

The oxidative stress measured as lipid peroxidation by MDA, CuONSp induced an increase in serum MDA level with high significant (*P <* 0.01) (97.52 ± 8.4 nmol/100 mg) and CuONF at 54.7 ± 4.6 nmol/100 mg compared to normal control (42.07 ± 4.4 nmol/100 mg). The elevation of CuONSp more than CuONF compared to control one. While in the measurements of antioxidant enzymes such as GSH, CuONSp induced a decrease in serum GSH activity with high significant (*P <* 0.01) (22.89 ± 1.3 U/g) and CuONF at 48.1 ± 1.4 U/g compared to normal control ones (55.34 ± 1.5 U/g). The inhibition to GSH activity by CuONSp is more than by CuONF compared to control (Fig. 7).

**Fig. 8:**
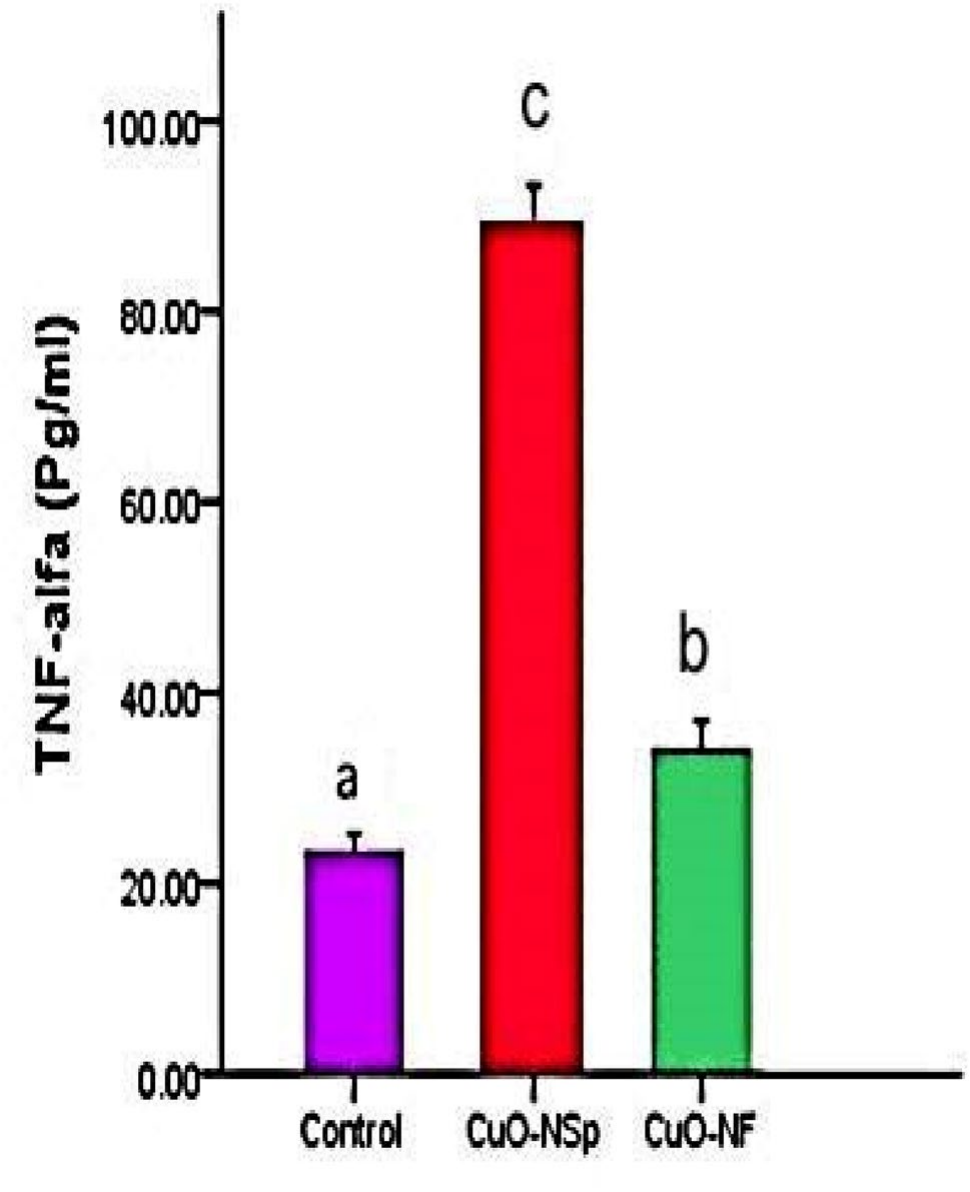
The toxic effects of CuONSp and CuONF on TNF-α level at the end of experiment. ^a,b,c^ indicated the difference or similarity between groups. Group with different superscript letters are considered significantly different (P < 0.05). There are six animals in each group

CuONSp induced increase in TNF-α level with high significant (*P <* 0.01) (88.57 ± 4.6 Pg/ml) and CuONF at (33.97 ± 3.1 Pg/ml) compared to normal control ones (23.23 ± 1.9 Pg/ml) at the end of experiment. As result, CuONSp induced an elevation in TNF-α level more than CuONF compared to control ones (Fig. 8).

### Histopathological study of liver and lung

**Fig. 9:**
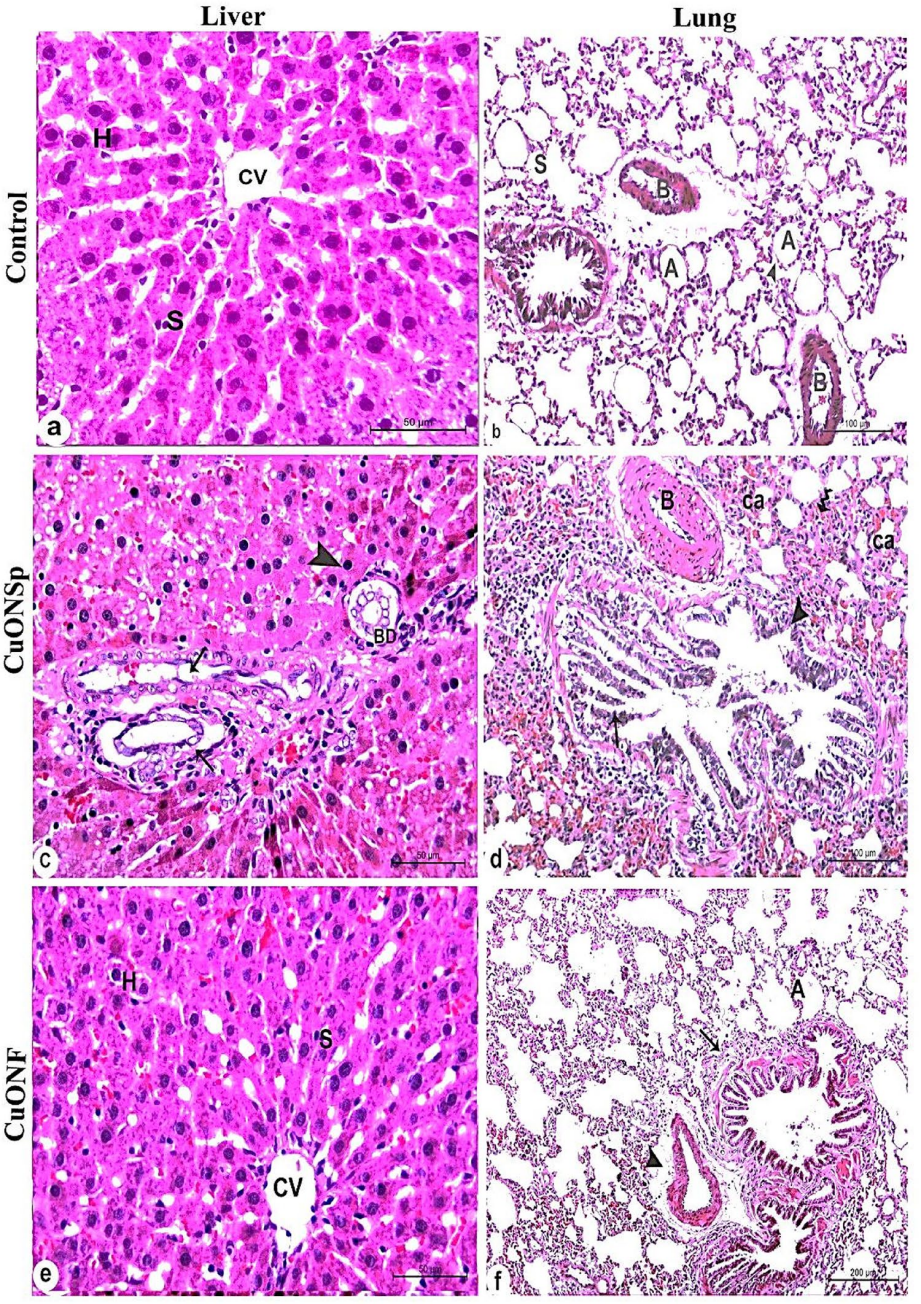
Photomicrographs of liver and lung of rats showing **a** normal structure of liver rats revealing; central vein (CV), sinusoids (S) and hepatocytes (H). **b** normal structure of lung rats showing; normal bronchiole (arrow), blood vessel (B), alveolar sac (s), alveoli (A), interalveolar septum (arrow head). liver rats sections treated with CuONSp showing **c** proliferation of bile ductules (BD), newly formed of bile ductules surounded with mononuclear leukocytic infiltration (arrows), and appearance some of pyknotic nuclei. **d** lung rats sections treated with CuONSp showing **d** thickened pulmonary blood vessel (B), marked hyperplasia of dilated bronchiole (arrow head), pyknotic nuclei (arrow), and focal areas of collapsed alveoli (Ca) and thickened interalveolar septum (wave arrow). While liver rats sections treated with CuONF. **e** showed improvements in structure of liver including the central vein (CV), hepatocytes (H) and sinusoids (S). Also, lung rats sections treated with CuONF. **f** illustrating improvement of blood vessel (arrow head), bronchiole (arrow) and alveoli (A). (H & E; Scale bar = 50 μm)

Microscopic examination of liver and lung control rats showed normal structure of both liver and lung (Fig. 9a, b) respectively. At the end of experiment, CuONSp rats sections showed proliferation of bile ductules, newly formed of bile ductules surounded with mononuclear leukocytic infiltration (Fig. 9c). In lung rats sections treated with CuONSp showed thickened pulmonary blood vessel, marked hyperplasia of dilated bronchiole, pyknotic nuclei, and focal areas of collapsed alveoli and thickened interalveolar septum (Fig. 9d). In liver rats sections treated with CuONF showed the structure of liver near to normal (Fig. 9e) and in lung rats sections treated with CuONF appeared to be normal (Fig. 9f).

### Immunohistochemical and morphometric analysis of liver and lung

**Fig. 10:**
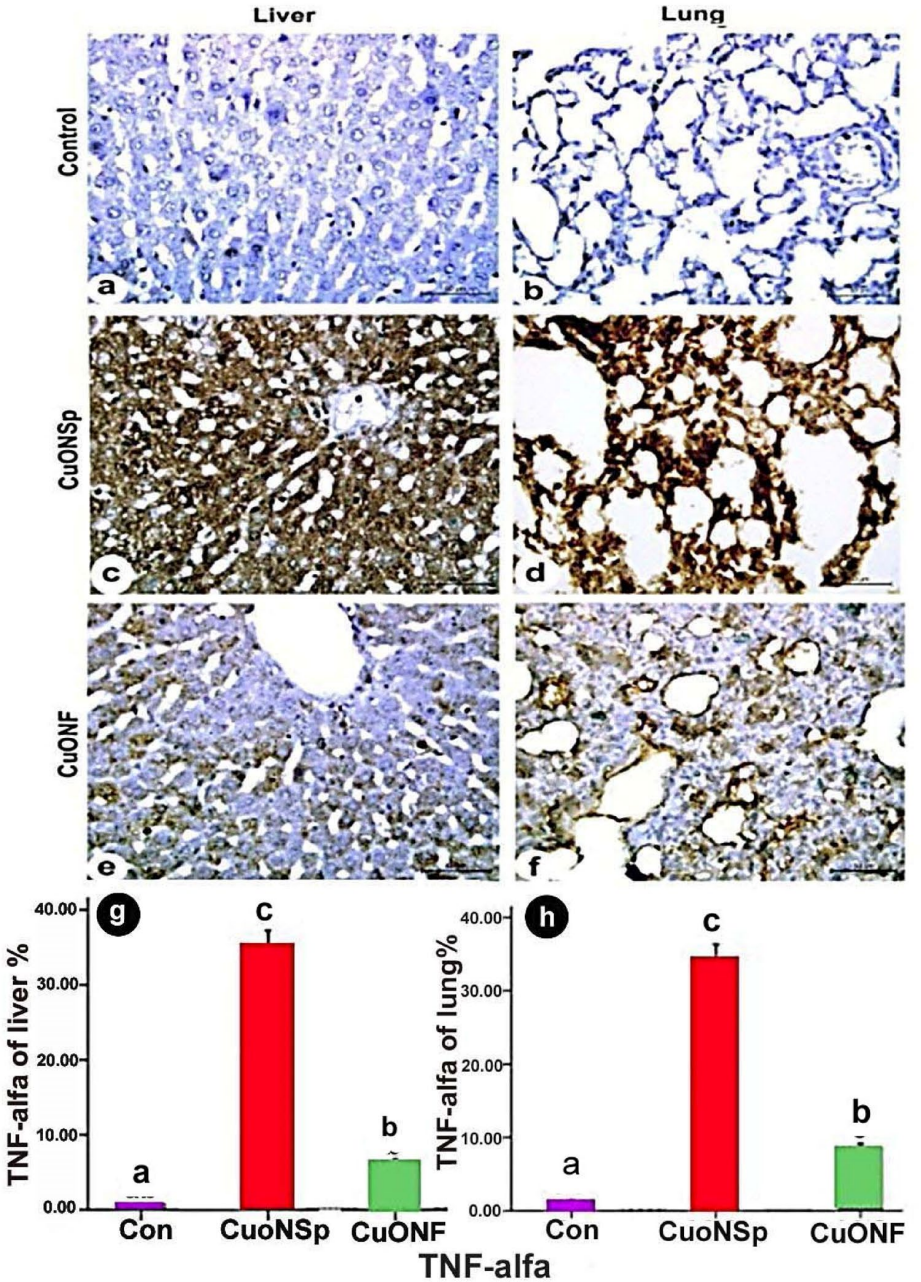
Photomicrographs of immunohistochemical expression of in liver and lung sections in CuONSp & CuONF treated groups showing **a, b** Negative immunohistochemical expression of TNF-α in control group in liver &lung respectively. **c, d** Strong positive immune-reactivity in cytoplasm of TNF-α in CuONSp treated group in liver and lung respectively. **e, f** Mild immno-expression for TNF-α of CuONF treated group in liver &lung respectively. (TNF-α; Scale bar = 50 μm). Bar charts showing the mean area of TNF-α immunostaining in liver and lung sections of CuONSp & CuONF respectively. ^a,b,c^ indicated the difference or similarity between groups. Group with different superscript letters are considered significantly different (P < 0.05). Groups with the same superscript letter did not present any significant differences

**Fig. 11:**
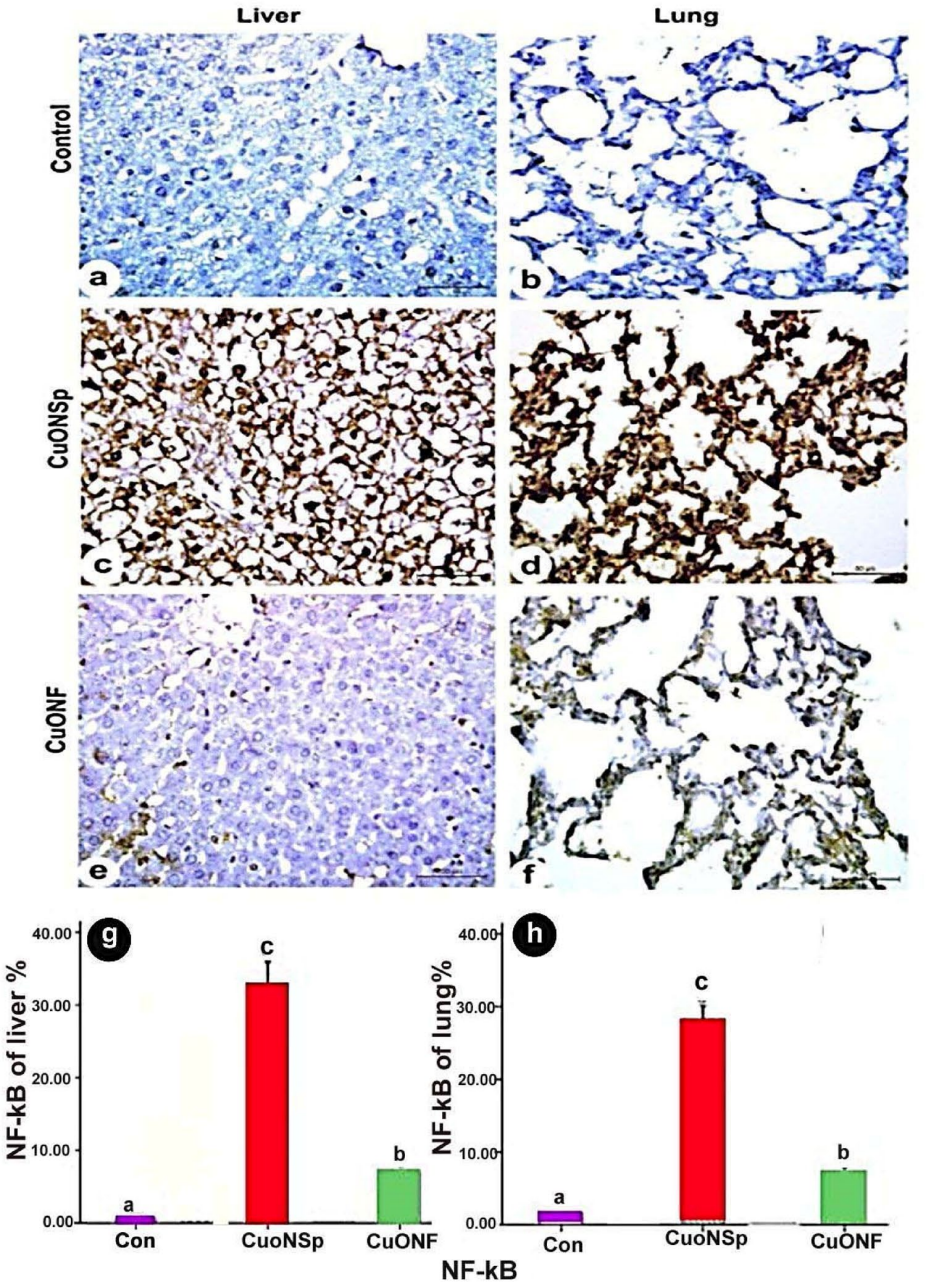
Photomicrographs of immunohistochemical expression of in liver and lung sections in CuONSp & CuONF treated groups showing **a, b** Negative immunohistochemical expression of NF-kβ in control group in liver &lung respectively. **c, d** Strong positive immune-reactivity in nuclei of NF-kβ in CuONSp treated group in liver and lung respectively. **e, f** few immno-expression for NF-kβ of CuONF treated group in liver &lung respectively. (NF-kβ; Scale bar = 50 μm). **g, h** Bar charts showing the mean area of NF-kβ immunostaining in liver and lung sections of CuONSp & CuONF respectively. ^a,b,c^ indicated the difference or similarity between groups. Group with different superscript letters are considered significantly different (P < 0.05). Groups with the same superscript letter did not present any significant differences

**Fig. 12:**
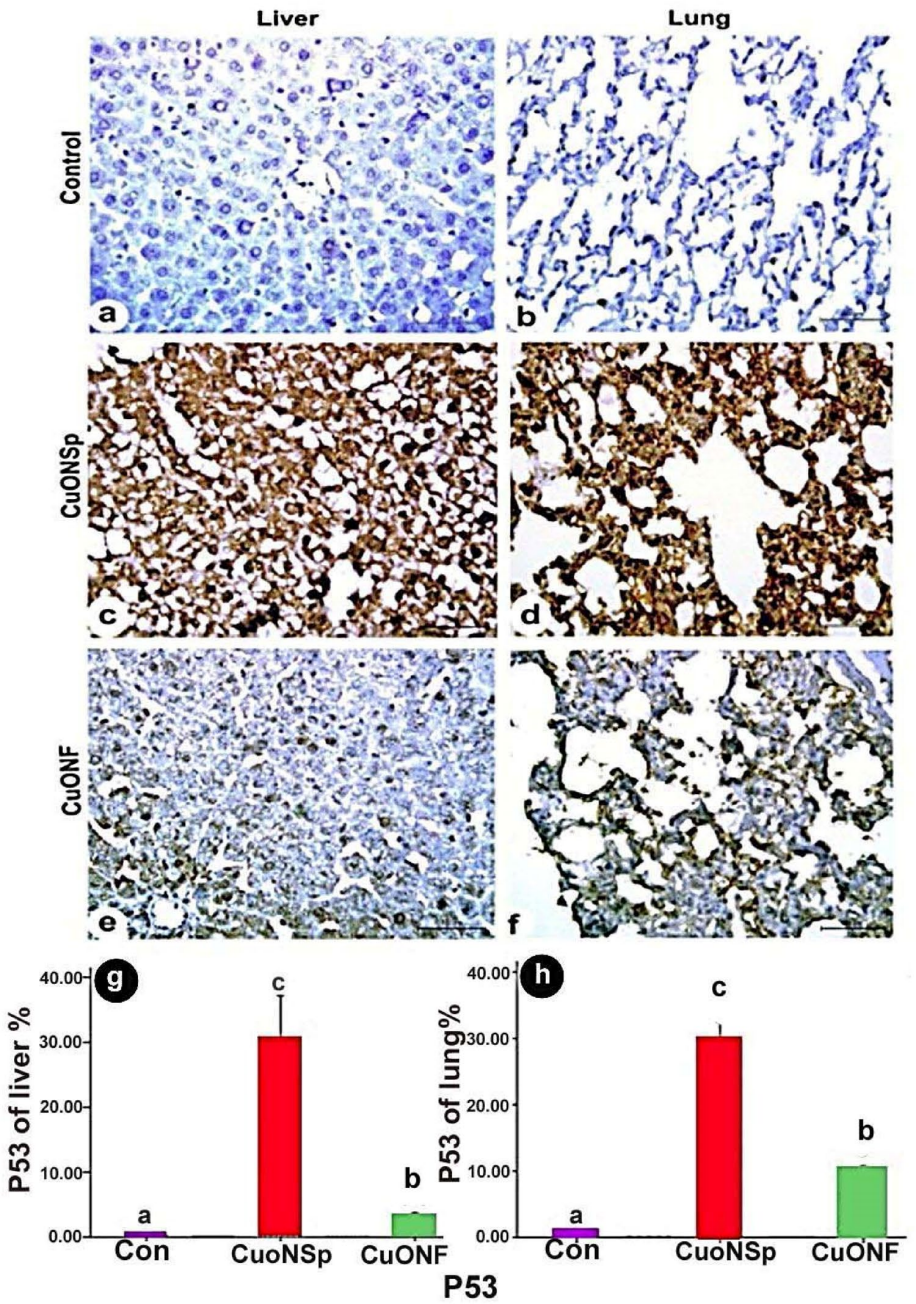
Photomicrographs of immunohistochemical expression of in liver and lung sections in CuONSp & CuONF treated groups showing **a, b** Negative immunohistochemical expression of P53 in control group in liver &lung respectively. **c, d** Strong positive immune-reactivity in nuclei of P53 in CuONSp treated group in liver and lung respectively. **e, f** few immno-expression for P53 of CuONF treated group in liver &lung respectively. (P53; Scale bar = 50 μm). **g, h** Bar charts showing the mean area of P53 immunostaining in liver and lung sections of CuONSp & CuONF respectively. ^a,b,c^ indicated the difference or similarity between groups. Group with different superscript letters are considered significantly different (P < 0.05). Groups with the same superscript letter did not present any significant differences

Immuno-histochemical expression of control liver demonstrated negative TNF-α (Fig. 10a), NF-kβ (Fig. 11a) and P53 (Fig. 12a) immune-expression. CuONSp treated group demonstrated strong positive immune-reactivity of TNF-α (Fig. 10c), NF-kβ (Fig. 11c) and P53 (Fig. 12c). CuONF-treated group revealed mild positive immune-reactivity of TNF-α (Fig. 10e), NF-kβ (Fig. 11e) and P53 (Fig. 12e). Liver of CuONSp-treated group induced a significant increase in mean area % of TNF-α (Fig. 10g), NF-kβ (Fig. 11g) and P53 (Fig. 12g) than CuONF-treated group compared to normal control ones.

Lung of control group showed negative TNF-α (Fig. 10b), NF-kβ (Fig. 11b) and P53 (Fig. 12b) immune-expression. CuONSp treated group of pulmonary tissue revealed intensive positive immune-reactivity of TNF-α (Fig. 10d), NF-kβ (Fig. 11d) and P53 (Fig. 12d). Lung CuONF-treated group illustrated few positive immune-reactivity of TNF-α (Fig. 10f), NF-kβ (Fig. 11f) and P53 (Fig. 12f). Lung CuONSp treated group induced an elevation in mean area % of TNF-α (Fig. 10h), NF-kβ (Fig. 11h) and P53 (Fig. 12h) with marked significant than CuONF compared to the normal control ones.

### Ultrastructural examination of liver and lung after exposure to CuONSp and CuONF

**Fig. 13:**
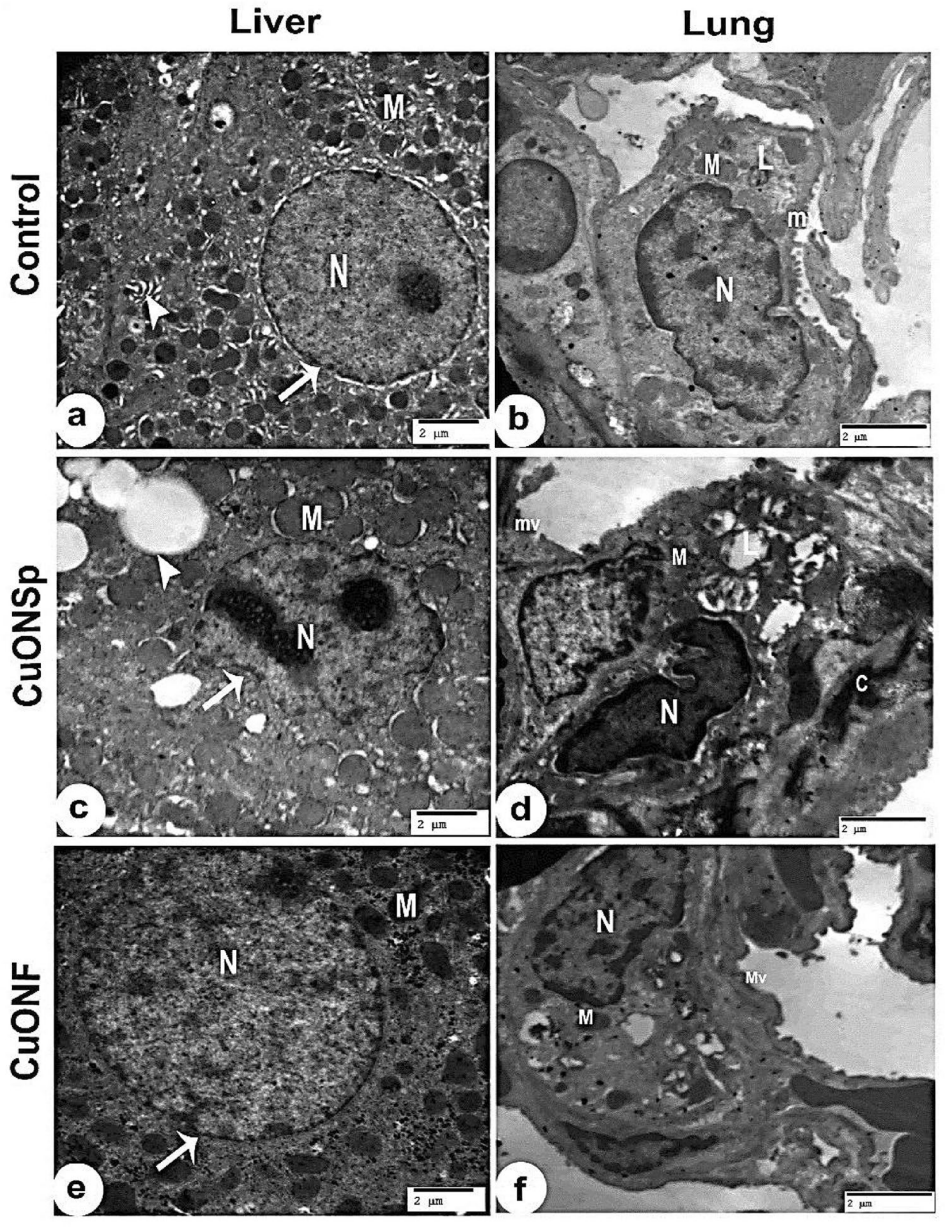
An Electron micrograph of rat liver; **a** Control group demonstrating a normal hepatocyte with rounded euchromatic nucleus (N), prominent nuclear membrane (arrow), regular rough endoplasmic reticulum (arrow head), and many round or oval mitochondria (M) with normal cristae. (TEM, Scale bar = 2 μm). **b** Electron micrographs of ultrathin section of control rat lung tissue shows nucleus (N) of pneumocyte type 2, lamellar bodies (L), around the nucleus, mitochondria (M), microvilli (mv) on the surface. (TEM, Scale bar = 2 μm). **c** Showing hepatocytes after treatment with CuONSp for 30 days, Nucleus (N) with irregular nuclear envelope (arrow), numerous swollen mitochondria (M) with ill-defined criste, variable-sized fat droplets (arrow head) (TEM, Scale bar = 2 μm). **d** Electron micrographs of ultrathin section of CuONSp treated group rat lung shows irregular pyknotic nucleus (N) of pneumocyte type 2 with empty lamellar bodies (L), degenerated mitochondria (M) lack of microvilli (mv). Notice the deposition of collagen fibres (C) (TEM, Scale bar = 2 μm). **e** hepatocytes treated with CuONF, revealing less toxicity compared to CuONSp group, the nucleus appear with normal chromatin pattern, nucleolus (N) and nuclear envelope (arrow), and mitochondria well-defined cristae (M) (TEM, Scale bar = 2 μm). **f** Lung treated with CuONF shows nearly normal nucleus (N), mitochondria (M) and intact microvilli (mv) on the surface (TEM, Scale bar = 2 μm)

Control liver demonstrated a normal structure (Fig. 13a) and lung control group revealed normal structure (Fig. 13b). Hepatocytes treated with CuONSp revealed nucleus with irregular nuclear envelope, numerous swollen mitochondria with ill-defined cristae and variable-sized fat droplets (Fig. 13c). Lung treated with CuONSp showed irregular pyknotic nucleus of pneumocyte type 2 with empty lamellar bodies, degenerated mitochondria lack of microvilli and deposition of collagen fibres (Fig. 13d). Hepatocytes treated with CuONF, revealed less toxicity compared to CuONSp group, the nucleus appeared with normal chromatin pattern, nucleolus and nuclear envelope, Mitochondria also appeared with well-defined cristae (Fig. 13e). Lung treated with CuONF showed nearly normal nucleus, mitochondria and intact microvilli on the surface (Fig. 13f).

## Discussion

Engineered NPs that have gained the most attention in other sectors have very little potential for large-scale agricultural applications (e.g., carbon nanotubes, nanosilver) (Aschberger et al. 2015) NPs are commonly used in nano-technology with a size < 100 nm (Linic et al. 2015). In our study, we used hydrothermal methods to produce two different shapes of CuONPs, including CuONSp and CuONF. Through synthesis of CuONSp, Cu (OH)_2_ precipitate is then converted in the nano-sphere under hydrothermal conditions; this result is in agreement with (Seo et al. 2013). Furthermore in preparation of CuONF, we used CTAB acts as a stabilizer for the synthesis of CuONF through hydrothermal process (Zhou et al. 2006).

In the XRD measurement of our study, the crystallite size was detected for CuONSp at 20.6 nm and recorded at 117.1 nm in CuONF. The absence of any other peaks indicates that the samples are pure. The increased sharpness of XRD peaks and the grain size observed for CuONPs using the relative intensity peak suggest that the particles are crystalline in nature. According to Mohammadyari et al. (2014) the grain size of CuONPs determined by the relative intensity peak was 50 nm, and an improvement in XRD peak sharpness indicates that the particles are in crystalline form. According to our findings, the alteration of NPs shape, which reduces the degree of interaction between cells and NPs. The lower contact with the cells, the most modified shape surface of the NPs changes, which explain that toxicity induced by CuONSp is more than by CuONF. Vasilakes et al. (2013) indicates that the shape of NPs is just as essential as their size in the passage of a drug through the body.

Our HRSEM measurements revealed the presence of aggromerated particles in two different shapes of CuONPs. Although the size of CuONSp was observed by HRTEM at rang 9 nm, it is under the category of quantum dots (QDs). CuONF has a mean range of 228 nm. As a result, CuONSp has smaller QD size in comparison to CuONF, CuONSp is the most toxic from CuONF. Oxidative stress is caused by the entry of QDs particles into cells and the production of free NPs ions (Ambrosone et al. 2012). There is proof that QDs penetration creases the fluidity of the cell membrane (Wang et al. 2012).

Our measurements of PDI to CuONSp detected at 0.43 and CuONF at 0.64. That CuONSp have the greatest particle stability and distibution from CuONF. These findings, confirmed by Masarudin et al. (2015) who stated that the PDI was used as an indicator of the stability and uniformity of nanoparticle formation. While, the lowest value in PDI the most stable in suspension. Our Zeta Potential measurements for CuONSp charge measured at − 50.9 ± 6.5mV and CuONF at − 47.2 ± 7.6mV. The surface charge of NPs plays an important role in their toxicity, since the interactions of NPs with biological systems are largely determined. More toxicity was caused by a significant amount of surface charge. As a result, the toxicity of CuONSp is the most toxic relative to CuONF. Sharifi et al. (2012) revealed that, the cationic surface charge, once internalized, functions as a proton sponge that inhibits normal lysosomal activity and initiates cell death.

It has been hypothesized that CuO nano-sized catalytic properties may be linked to part of the ROS generation. Over production of ROS will disturb the balance of the oxidative / antioxidant mechanism of the liver, resulting in lipid peroxidation through the creation of ROS and MDA and hepatocyte apoptosis, which may be closely linked to the reduction of antioxidant enzymes (Brown et al. 2001). The decrease in cell viability observed could be due to an increase in oxidative stress after exposure to CuONPs (Abudayyak et al. 2020). In the current study, CuONPs were orally treated to rats at a dose 50 nm at two different shapes; CuONSp induced an elevation MDA level more than CuONF compared to control. Also, CuONSp induced GSH inhibition activity of more than CuONF in compared to control. The toxicity of metal NPs and the excessive growth of ROS, oxidative stress have been involved and induced apoptosis may be one of the possible toxicity mechanisms of NPs (Benameur et al. 2015). Also, Rossner et al. (2020) said that employed next generation sequencing protocols and determined gene expression changes in the lungs of mice exposed to inhalation of CuO NPs. In rats intoxicated with CuONPs, hepatic levels of MDA were significantly elevated compared to normal rats and a decrease in activity of GSH in the liver relative to normal rats (Abdelazeim et al. 2020; Boyadzhiev et al. 2021) reported that the up-regulation of DNA was presented in a recent transcriptomics, where mouse lung epithelial cells were exposed to varying doses of CuO NPs for different periods of time.

Our biochemical analysis of CuONSp and CuONF groups revealed that CuONSp induced an elevation in AST, ALT &GGT activities with a high significant than CuONF. These in accordance with Rabia et al. (2019) who suggested that CuONPs displayed histological changes which correlated with the recorded increased in liver AST and ALT. According to Pietrofesa et al. (2021) inhalation of CuO NPs causes an inflammatory reaction that damages cells and lung tissues. In the current microscopic observations, the liver of CuONSp treated rats showed proliferation of bile ductules, newly developed bile ductules surrounded by mononuclear leukocytic infiltration at the end of experiment. However, in CuONF treated rats, liver nearly appeared with normal structure. In CuONSp treated lung rats, thickened pulmonary blood vessel, pronounced hyperplasia of dilated bronchiole, pyknotic nuclei, and focal areas of collapsed alveoli and thickened interalveolar septum. However, CuONF treated rats; lung appeared near to normal structure. The amount of Cu in lungs and liver was significantly increased after six weeks of CuO NPs exposure (Tulinska et al. 2022; Dumková et al. 2017) found that major changes in the liver occurred during sub chronic exposure to lead oxide nanoparticles (PbO NPs), primarily manifested by hepatocyte enlargement and hydropic degeneration. Also, inhalation of PbO NPs induced significant alterations in lung morphology following six weeks exposure. Lungs of these animals displayed mild hyperaemia, congested capillaries, swollen pulmonary septa, haemostasis in basal parts of lobes with involvement of several siderophages (macrophages with pigment hemosiderin) and the presence of alveolar emphysema in certain parts of the lungs. Valentini et al. (2019) reported that Titanium oxide nanoparticles (TiO_2_-NPs) have entered the liver *via* blood circulation following intraperitoneal injection. Kupffer cells, possibly phagolysosomes, located in hepatic sinusoids as well as in the periphery of the portal tract, were internalized with NP aggregates. These aggregates emerged as spherical inclusions of the refringent. NPs were also present in some hepatocytes in dense cytoplasmic inclusions.

Our immunohistochemical staining of TNF-α, NF-kβ & P53 in liver and lung sections revealed, strong positive expression of TNF-α, NF-kβ & P53 immune-expressions in CuONSp treated rats, but mild expression was observed in liver and lung sections CuONF-treated rats. Moreover, in the present examination, CuONSp caused a rise in serum TNF-α level more than with CuONF (Abdelazeim et al. 2020). The inflammatory cascade includes profibrotic mediators that have been implicated in fibrosis pathogenesis, such as TNF-α. Cells are known to reverse the overwhelming oxidative stress response through increased expression of cytokines such as TNF-α, kinase activation, and phosphatase inhibition, thus affecting the cascade of phosphorylation (Genestra 2007). Several metal oxide NPs increase the activation of NF-kβ pathways (Smith et al. 2001). During stressful conditions, such as ROS-mediated DNA damage, p53 can protect the genome. In order to allow time for damage to be repaired, the primary role of the genome is to cause cell cycle arrest. The cell responds by switching to apoptosis if the DNA damage cannot be repaired (Li et al. 2012).

In our ultrastructural studies, hepatocytes treated with CuONSp revealed nucleus with irregular nuclear envelope, numerous swollen mitochondria with ill-defined cristae, variable-sized fat droplets. Lung treated with CuONSp showed irregular pyknotic nucleus of pneumocyte type 2 with empty lamellar bodies, degenerated mitochondria lack of microvilli and deposition of collagen fibres. While, hepatocytes treated with CuONF, revealed less toxicity compared to CuONSp- treated group, the nucleus appear with normal chromatin pattern, nucleolus and nuclear envelope. Also, appear with mitochondria well-defined cristae. Also, lung treated with CuONF showed nucleus near to normal pneumocyte type 2, mitochondria in cytoplasm with microvilli on the surface. Semisch et al. (2014) demonstrated that the damage of mitochondrial membrane of liver cells can be generated by direct interactions with CuONPs or by ROS release, leading to apoptotic enzyme discharge. ROS generation induced by NPs causes harmful to DNA, proteins and organelles, including mitochondria. Damaged mitochondria cause intrinsic and subsequently extrinsic apoptotic pathways to be activated (Ou et al. 2016; Roda et al. 2019) revealed that the early initiation of inflammation has been detected, as evidenced by the increased presence of various inflammatory cells, including multiple activated Kupffer cells and phagosomes. Furthermore, a fibrotic reaction has been demonstrated by an evident deposition of collagen fibres. Gaharwar et al. (2019) suggested that exposure to NPs induced vacuolated mitochondria and lung tissue damage in compared to the control tissue. The surfactant in the alveoli encased by NPs enhanced tubular myelin production was associated with increased surfactant production by alveolar epithelial type 2 cells (PII).

## Conclusion

CuONSp and CuONF were synthesised using hydrothermal methods in our research. CuONSp and CuONF were found to be crystalline in XRD measurements. The smaller the particles, the higher their toxicity. CuONSp has smaller particles than CuONF, so it is more toxic than CuONF. The PDI was used to determine the stability of NPs, a lower value indicates more stability and more toxicity. The value of PDI for CuONSp is lower than for CuONF. So, it is more toxic than CuONF. Also, the toxicity was determined by the amount of surface charges on nanoparticles. CuONSp has more charges than CuONF, so it has more toxicity. Around our biochemical alternations induced by CuONPs. There were an elevation in MDA level, AST, ALT & GGT activities. The Bax level is more in CuONSp than CuONF. As well as, an inhibition in GSH, SOD and CAT activities as well as in Bcl2 level induced by CuONSp < CuONF. Histopathological and ultrastructural studies in liver & lung tissues induced marked alternations by CuONSp > CuONF. Our immune-expression of TNF-α, NF-Kβ & P53 in liver and lung sections in various treatment groups, showing intense positive immune-expression of TNF-α, NF-Kβ & P53 expressions in the CuONSp treated group, mild positive TNF-α, NF-Kβ & P53 expressions in the CuONF treated group. Therefore, according to the present results CuONSp & CuONF and the toxicity induced in different studies, CuONF is better in use than CuONSp in agriculture applications due to its lower toxicity. The present study suggests that further studies are required to investigate its safety and toxicity compared to other form.

## Electronic supplementary material

Supplementary Material 1

## Data Availability

The datasets generated during and/or analyzed during the current study are available from the corresponding author on reasonable request.
