# Supplementary material for: Comparative toxicological evaluations of novel forms nano-pesticides in liver and lung of albino rats
Source: J Mol Histol. 2023 Mar 31;54(2):157–72. doi: 10.1007/s10735-023-10115-y (PMC10079706; doi:10.1007/s10735-023-10115-y)
Supplement: Supplementary file 1 — Supplementary Material 1 [file 10735_2023_10115_MOESM1_ESM.docx]

**Table S1: Illustrate our result of X-ray diffraction (XRD) pattern of CuONSp match with those of CuONP card number** **96-901-5925 .**

| d-spacing [Å] | Pos. [°2Th.] | hkl)) | Rel. Int. [%] | Micro Strain only [%] |
| --- | --- | --- | --- | --- |
| 2.75770 | 32.4677 | 313¯ | 12.17 | 0.44 |
| 2.53039 | 35.4767 | 002 | 100.00 | 0.61 |
| 2.33609 | 38.5389 | 111 | 97.76 | 0.59 |
| 1.96452 | 46.2116 | ¯112 | 2.36 | 0.61 |
| 1.86668 | 48.7866 | ¯202 | 20.92 | 0.43 |
| 1.71214 | 53.5228 | 020 | 5.01 | 0.52 |
| 1.58895 | 58.0494 | 202 | 8.85 | 0.55 |
| 1.50956 | 61.4212 | ¯113 | 15.49 | 0.14 |
| 1.41011 | 66.2847 | 022 | 14.61 | 0.60 |
| 1.37907 | 67.9768 | 220 | 12.35 | 0.32 |
| 1.30865 | 72.1879 | 311 | 3.40 | 0.42 |
| 1.27098 | 74.6833 | 004 | 5.82 | 0.40 |
| 1.16742 | 82.6576 | ¯313 | 3.87 | 0.70 |

The data revealed the formation of CuO nanostructures in monoclinic symmetry with space group C12/c1.

**Table S2: Illustrate our result of X-ray diffraction (XRD) pattern of CuONF match with those of CuONP card number** **96-901-5823.**

| d-spacing [Å] | Pos. [°2Th.] | (hkl) | Rel. Int. [%] | Micro Strain only [%] |
| --- | --- | --- | --- | --- |
| 2.76111 | 32.4264 | 110 | 15.17 | 0.20 |
| 2.52919 | 35.4942 | ¯111 | 100.00 | 0.10 |
| 2.33001 | 38.6435 | 111 | 89.08 | 0.13 |
| 1.96554 | 46.1863 | ¯112 | 1.63 | 0.30 |
| 1.86794 | 48.7517 | ¯202 | 17.78 | 0.28 |
| 1.78236 | 51.2573 | 112 | 1.41 | 0.36 |
| 1.58546 | 58.1893 | 202 | 7.15 | 0.23 |
| 1.50836 | 61.4757 | ¯113 | 9.63 | 0.29 |
| 1.41196 | 66.1868 | ¯311 | 10.29 | 0.12 |
| 1.37805 | 68.0344 | ¯221 | 9.05 | 0.25 |
| 1.30649 | 72.3260 | 311 | 3.60 | 0.08 |
| 1.26306 | 75.2328 | ¯222 | 4.51 | 0.19 |
| 1.16832 | 82.5798 | 222 | 2.08 | 0.39 |

The data revealed the formation of CuO nanostructures in monoclinic symmetry with space group C12/c1.
